# Application of Chromosome Conformation Capture Method for Detection MYC/TRD Chromosomal Translocation in Leukemia Cell Line

**DOI:** 10.18502/ijhoscr.v14i3.3729

**Published:** 2020-07-01

**Authors:** Moloud Absalan, Mohammad Hossein Ghahremani, Zahra Jabbarpour, Roya Karimi, Shilan Shafei, Reza Heidari, Mostafa Akbariqomi, Gholamreza Tavoosidana

**Affiliations:** 1 Department of Molecular Medicine, School of Advanced Technologies in Medicine, Tehran University of Medical Sciences, Tehran, Iran; 2 Department of Pharmacology and Toxicology, School of Pharmacy, Tehran University of Medical Sciences, Tehran, Iran; 3 Department of Tissue Engineering and Applied Cell Sciences, School of Advanced Technologies in Medicine, Tehran University of Medical Sciences, Tehran, Iran.

**Keywords:** Chromosomal rearrangements, Chromosome conformation capture, MYC/TRD, Inverse-polymerase chain reaction

## Abstract

**Background:** Chromosomal breakpoints are the most common cause of hereditary diseases and cancers. Today, many standard clinical methods such as cytogenetic and PCR based techniques are used which have limitation regarding detection resolution. Chromosome conformation capture is a method for detecting gene proximity and chromosomal rearrangements.

**Materials and Methods:** In this study, SKW3 cell line was used for detecting t(8;14)(q24;q11) using a 3C-based technique. SKW3 cell line was used for 3C library preparation. For Inverse PCR, two regions were selected in upstream and downstream of the viewpoint locus on chromosome 8-MYC gene based on EcoRI restriction sites. The captured sequence with intra-chromosomal interaction between chr8-c-MYC and chr14-TRD was selected for the translocation PCR primer design.

**Results:** The DNA fragment captured in 3C PCR showed a specific TRD sequence translocated downstream of the MYC gene. Translocation PCR demonstrated the existence of (8; 14) (q24; q11) MYC /TRD in both library and genomic DNA.

**Conclusion:** This result demonstrated 3C- based method could be used as a useful low-cost easy operating technique in chromosomal rearrangements detection. In this study, the integration of whole genome library monitoring and PCR method was used as a high- through put method in chromosomal breakpoints detection.

## Introduction

 Chromosomal translocations are considered as the common incidence in human cancers, especially in hematopoietic and lymphoid malignancies^   1 ^^.^ Balanced chromosomal rearrangements occur from 1/500 to 1/625 incidence range^   2 ^. DNA double strand breaks at two loci and joining the ends of DNA breaks in an illegitimately way are two main biological processes which could lead to the formation of chromosomal translocation. The sequences of events lead to influencing phenotype due to the proximity of the proto-oncogene with cis regulatory elements. The over expression of proto-oncogene is the main scenario in Burkitt lymphoma in which t(8;14) results in MYC juxta positioning to immunoglobulin heavy chain (IGH) regulatory elements. In lymphocytes, Chromosome 8 is close to chromosome 14 spatially^3^. Chromosomal breakpoints with the involvement of T- cell receptor are one of the most common incidences in T-cell acute lymphoblastic leukemia patients^   4 ^. These translocations are associated with V (D) J fragments recombination. This event may lead to activation of the proto-oncogene placing nearby the cis regulatory elements which were carried through these recombined fragments ^   5 ^. The understanding of their incidence in genome may help us to develop new approaches for early detection and therapy of leukemia. Chromosome conformation capture (3C) technique developed by Dekker et al. in 2002 is a powerful method for conducting studies regarding genes proximity and epigenetic ^   6 ^. As it was described in Schilit et al. study, 3C-PCR is an inexpensive and efficient proximity ligation-based approach. 3C-PCR targets the allele of a variable locus in a chromosomal rearrangement on a derivative chromosome by a simple nested PCR strategy on 3C libraries. It does not need specific primers and the amplification products with variable sizes due to different ligation products through 3C library preparation^   7 ^. Chromosome conformation capture prepares a pool of genomic-wide Intra– and inter-chromosomal interactions. The 3C -based methods are consisted of chromatin fixation using fixative agents such as formaldehyde , restriction enzymes digestion, ligation of cross-linked fragments under optimized condition and reversing the cross-linked chromatin which result in 3C library conformation^   8 ^^.^ In the 4C (Circular Chromosome Conformation Capture), by using specific viewpoint primers, all the fragments contacting or have adequate proximity to the locus of interest will be captured through Inverse PCR amplification. The captured sequences can be analyzed sequencing or microarray. The 3C -based methods can reveal the interaction between genes and their regulatory elements close or located on other chromosomes^   9 ^. The Chromatin organization influences gene expression through altering the proximity of special locus to regulatory elements ^   10 ^. Although 3C- based methods investigate the 3D organization of a whole genome originally , the data collected from such methods can also be used for detecting chromosomal rearrangements, genome sequencing and haplotyping^   11 ^. Based on this knowledge, 4C was used to identify a new oncogenic translocation in childhood leukemia ^12^^,^^13^ . Today, chromosome banding fluorescent in situ hybridization (FISH) and array comparative genome hybridization (CGH) are used for detecting chromosomal aberrations, but none of them are capable to detect the low number of base- pair resolution. On the other hand, NGS (Next Generation Sequencing) and WGS (Whole Genome Sequencing) which could detect the chromosomal breakpoint are regarded as the best tools for mapping chromosomal rearrangements. However, considerable sequencing depth, cost and elimination of false positive caused by sequencing errors make these methods unsuitable for routine clinical detection^14^. In this study , SKW-3 T-cell leukemia (derivative of KE-37) cell line was selected as a model for chromosomal breakpoint which carries t(8;14)(q24;q11).The aim of this study is to find MYC /TRD (T-Cell Receptor Delta) in SKW3 cell line using a 3C- based method which is a cheap , high through- put PCR based technique.

## MATERIALS AND METHODS

**Cell culture and characterization**

SKW3 cells purchased from cell bank of Pasteur institute, Iran were cultured with Gibco RPMI 1640 medium supplemented with 10% FBS (Fetal Bovine Serum) and 1% penicillin- streptomycin .The cells were incubated at 37 °C with 5% CO_2_^.^ The cell culture media was replaced 3 times a week. The SKW3 cell line can be identified by differential expression of cell surface(CD) markers, including CD2+, CD3- , CD4 +, CD7+ and CD13- ^15^^, ^^16^ . 1x10^6^ fresh dividing cells were conjugated with specific antibody and were given to FACS in flow cytometry workflow and were analyzed using FlowJo software.

**3C library preparation**

6x10^6 ^cells were collected through centrifuging and were used for the 3C library preparation and genomic DNA extraction. Library preparation was operated on skw3 cell line suspended in the 3C buffer (50 mM Tris-HCl pH 8.0; 50 mM NaCl; 10 mM MgCl2; 1 mM DTT). Formaldehyde 37% added as a fixative reagent to make the crosslinks in the final concentration of 1%. The reaction was blocked by 1M Glycine incubated on ice. DNA digestion was done by adding 150 units (U) of EcoRI enzyme serially for a total of 450 U for an overnight. The digestion reaction was inactivated by adding Sodium dodecyl sulfate and Triton X-100. T4 ligase (30U/µl) and ATP (Fermentas R0441) was added to ligate the fragments with the most proximity. In order to reverse the crosslinked DNA, PK buffer (5 mM EDTA pH 8.0; 10 mM Tris-HCl pH 8.0; 0.5% SDS) and proteinase K (PK 20mg/ml) were added to the 3C library mixture and was incubated during 1 h at 50°C. The 3C library was purified by phenol chloroform protocol. The purified 3C library from SKW3 cell line was analyzed through gel electrophoresis and was used for quantification using Nano drop and was aliquoted for the inverse PCR^   17 ^.

**Inverse PCR**

**Figure 1:**
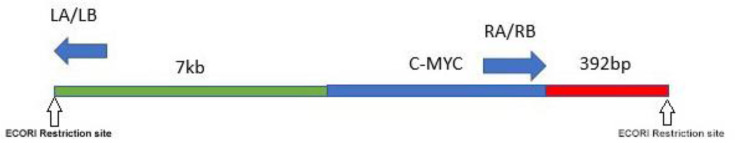
Two regions were selected in upstream (7Kb) and downstream (392 bp) of the viewpoint locus on chromosome 8-MYC gene based on EcoRI restriction sites for Inverse PCR primer design.

For the optimization of Inverse PCR, two regions were selected in upstream and downstream of the viewpoint locus on chromosome 8-MYC gene based on EcoRI restriction sites (Table 1, Figure 1). The same concentration of 3C library and genomic DNA from SKW3 cell line were used for the PCR assay. In the first set of PCR, the reaction consisted of first set of primers, leftA /RightA and total volume of 25µl. PCR products of the first reaction were 1:10 diluted and were used as the template for the second PCR using the second set of primers left B/Right B. PCR reactions were run under the following conditions: First PCR : 5 min at 95 Cº, following by 40 cycles of 30 sec 95 Cº,1 min 57 Cº,1:30 min 72 Cº and 10 min 72 Cº. Second PCR: 5 min 95 Cº following by 35 cycles of 30 sec 95 Cº, 45 sec 58 Cº, 45 sec72 Cº and 5 min72 Cº. PCR products of the second PCR from skw3 cell lines 3C library and genomic DNA were analyzed through gel electrophoresis, were purified using Qiagens̓ gel purification kit (Cat No./ID: 28704) according to the manufacturer’s instruction. The fragments were sequenced by Bioneer Company, South Korea using standard Sanger sequencing service cat.no.S-3010-1.

**Table 1:** Primers for Inverse PCR

Left A	5'-ATGAGGTTCTCCATTATGAGCTTG-3'
Right A	5'-ATCATTGAGCCAAATCTTAAGTTGTG-3'
Left B	5'-GTTCTCCATTATGAGCTTGGAATTC-3'
Right B	5'-GGCAAATATATCATTGAGCCAAAT-3'

## Results

**Cell culture and characterization**

**Figure 2:**
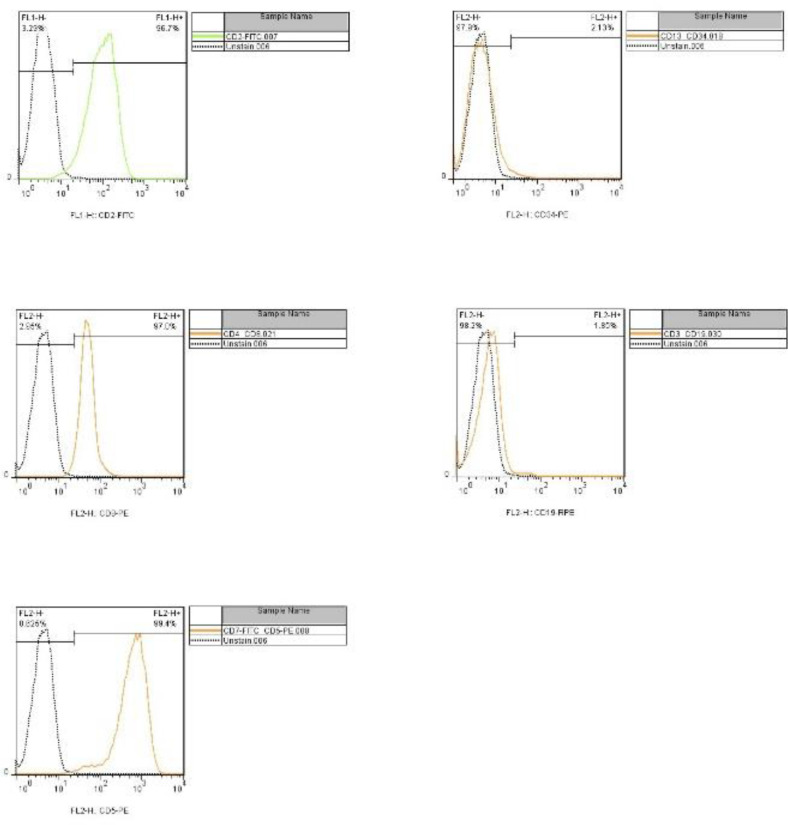
Analysis of Flow cytometry revealed that the surface markers profile is identical to what was expected in SKW3 cell line ATCC.

SKW3 cells were examined for detecting cell surface markers. Results are shown in Figure 2. Analysis of SKW3 cell line flow cytometry demonstrated that the cells were positive for CD2, CD4 and CD7 and negative for CD13 and CD3.

**3C Library preparation**

**Figure 3:**
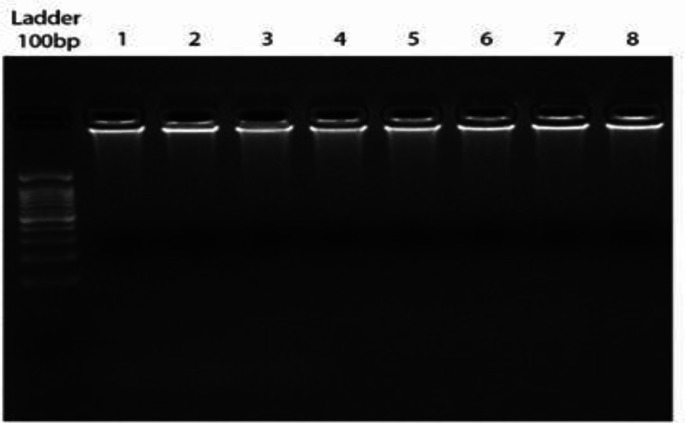
The integrity of 3C libraries was shown with a distinct smear band on gel electrophoresis.

The purified 3C library and the extracted genomic DNA from SKW3 cell line were evaluated in terms of concentration and purity. The integrity of libraries was shown with a distinct smear band for each library on gel electrophoresis (Figure3). The concentration of purified library was between 1-3µg/µl and the 260/280 nm ratio was equal to 1.8-2. 

**Inverse PCR**

**Figure 4:**
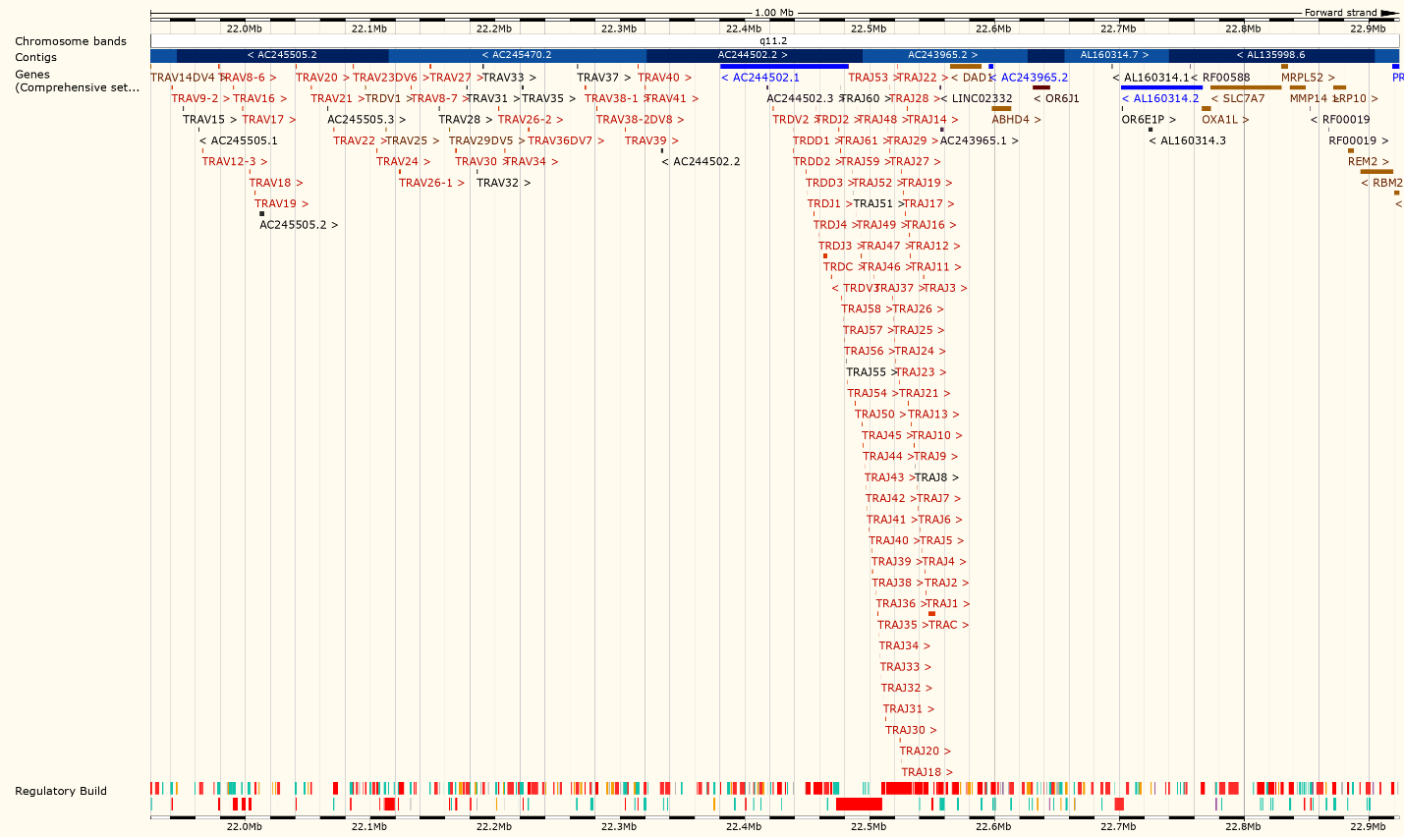
Ensemble database of No 8 sample similarity to AC244502.1 gene bank ID -Chromosome 14-TRD locus.

**Figure 5:**
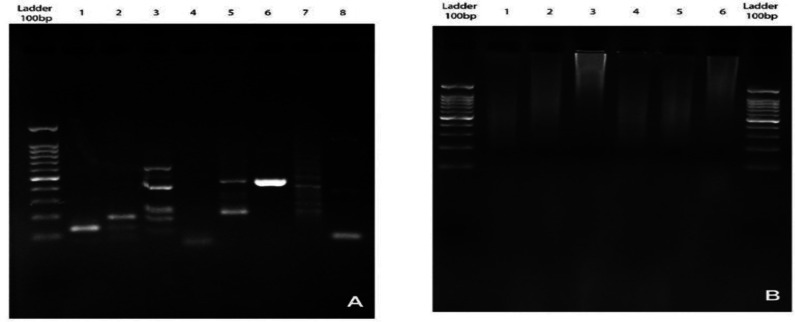
The results of 3C library inverse PCR showed variable sizes of captured fragments due to chimeric products in 3C library preparation (A). Sample NO 8 was selected for translocation PCR primer design which has the acquirable intra-chromosomal interaction. There were no specific DNA bands obtained from genomic DNA Inverse PCR (B).This could be due to the long distance between the viewpoint locus and juxtaposed sequences in the genomic DNA.

The PCR products of SKW3 pooled libraries and extracted genomic DNA were run on 2% agarose gel. The results indicated that there were no specific bands detected in genomic DNA through Inverse PCR amplification. Our results obtained from both first and second PCR amplification showed DNA smears indicated that the PCR primers could not anneal as it was desired (Figure5, B). 3 sets of 3C libraries were used for performing inverse PCR using two sets of primers. Results from the inverse PCR of 3C libraries showed specific bands with variant sizes from 100bp (Base pair) to 1kb (Figure5, A). The sample number "8" from 3C library was selected for the translocation PCR primer design on juxtaposed sequence with the size about 1000bp. The 517 bp of this fragment is shown in Table 2 and Figure 4. This sequence represented as the Juxtaposed fragment from chromosome 14, and therefore selected for the Reverse Primer design in Translocation PCR. Forward Primer also designed using sequence on 3' end of MYC locus 127741434-127741825 on chromosome 8 (Table 3).

**Table 2:** Sequence data for Fragment NO .8

ACAGTCTTTCATTCTTTACCAGTCTCAGGTATTCCTTTATAGCAACACAAACAGACTAAGACATTCCTGTACAGAAAGTC TGCCAACTGCTGATCTAGGAAAACTGACTGTGGCATCTGCTCCCTTAACCACTGCCCTAGACTGCCTCTGGGGCCCTCAG CTAGCAGTGATGATAAATGCTGGTGTGTTTTGACCCTCCAACAAGATGCATCCTTTGGAGAGAAGGGACATGACCCTTCT TAGGTACAGTTTTTTTTGTTTTTGTTTTTTGTTTGTTTGTTTGTTTTGAGATGGAGTTTTGCTCTTGTTGCCCAGGCTGG AGTGCAATGACACAATCTCAGTTCACCACAACCTCTGCTTCCTGGGTTCAAGCAATTCTCCTGCCTTAGCCTCCTGAGTA GCTGGTATTACAGGCATGCACCACCACATCCCGCTAATTTATTTATTTATTTTTTTTATTAGAAACGGGGTTTCACCTTG TCTGTTGACCAAGCTGATCTTGAAGTTCGATCGCAGG

**Table 3:** primers for Translocation PCR

F-primer	5'-GGAATGGCAGAAGGCAGGTGAGAAGGT-3'
R-primer	5'-GACACAGCAAGACCCCGTCTCATGAAG-3'

**Translocation PCR**

**Figure 6:**
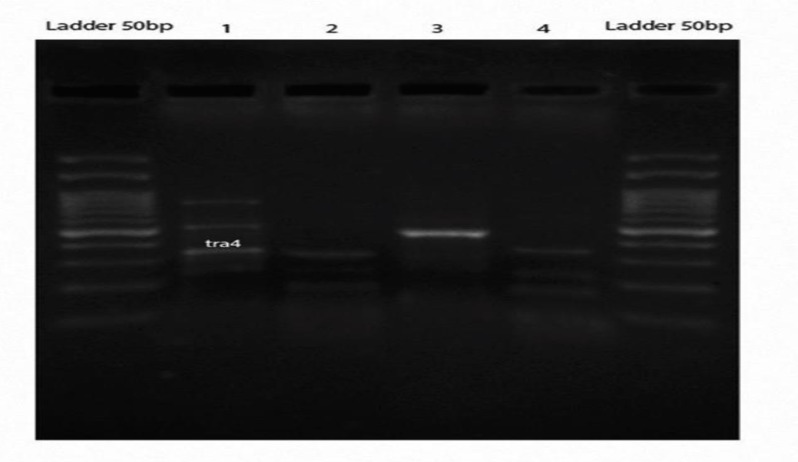
Translocation PCR performed on both 3C library and genomic DNA of SKW3 cell line. Sample 1 and 2 were indicated as 3C library and sample 3 and 4 were indicated as Digested/Ligated genomic DNA. The 184 bp (tra4) band from juxtaposed MYC /TRD was amplified in both 3C library and DNA using specific primers.

**Figure 7:**
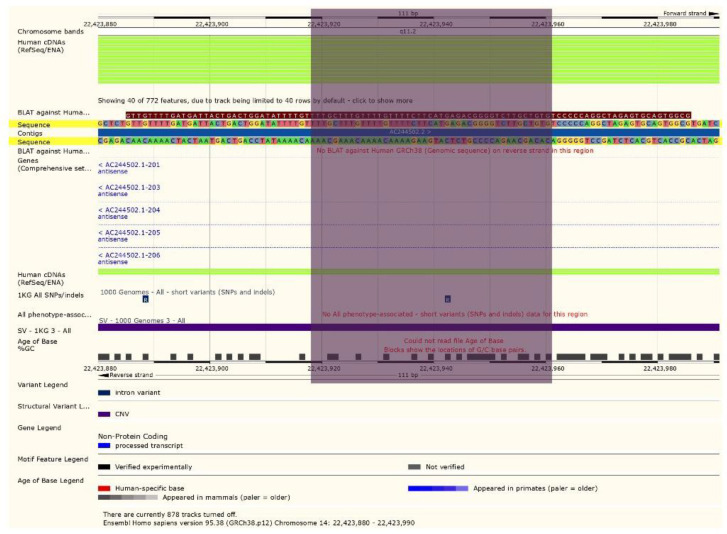
A 45bp fragment on Chr 14: 22423917 to 22423962 was identical to TRD locus <AC244502.1-201-6.

The result of sequencing both SKW3 3C library and digested/Ligated genomic DNA in translocation PCR assay showed a 184 bp DNA fragment (Table 4, Figure 6 ,7,8 ) that is identical to MYC gene chr 8:127741687 to 127741825. The MYC sequence was continued with 45bp of TRD gene on chr 14: 22423917 to 22423962. This data demonstrated the existence of (8; 14) (q24; q11) MYC /TRD in both library and genomic DNA. Results showed DNA fragment “tra4” is identical to the predicted translocation breakpoint sequence. Also, the result of aligning the “tra4” sequence and MYC reference gene showed complete similarity between the first segment and MYC gene. 

**Table 4:** Tra4 sequence indicated MYC /TRD juxtaposed fragment

>tra4-tra-F CCGAAGGTAGCAGGAGAACAGAGGTCAAGGTAGCAGTTAAGTACACAAAGAGGCATAAGG ACTGGGGAGTTGGGAGGAAGGTGAGGAAAAAACTCCTGTTACTTTAGTTAACCAGTGCCA GTCCCCTGCTCACTCCAAACCCAGCCTGATTTAAAAAAAACCTTCATGAGACGGGGTCTTGCTGTGTCA

**Figure 8:**
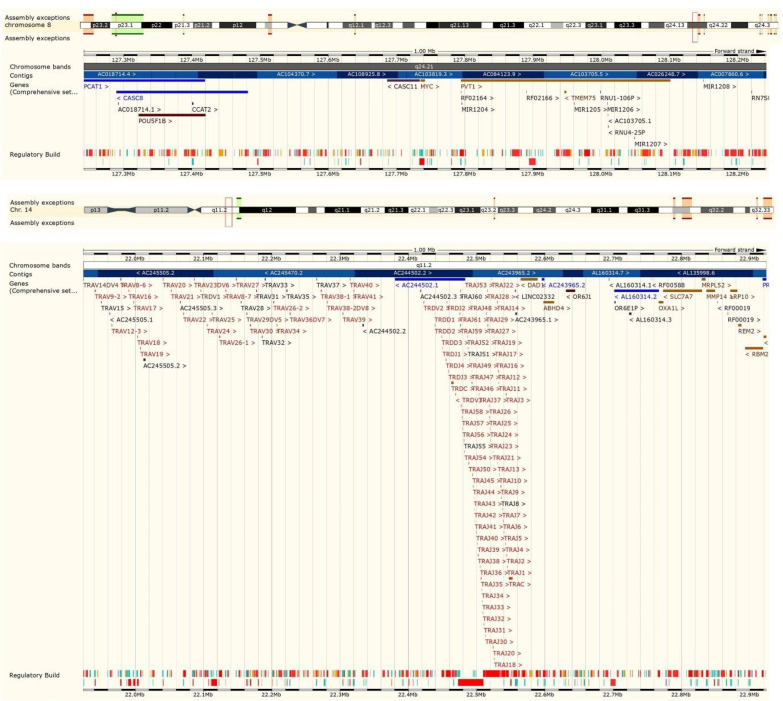
Ensemble data supports the similarity of the DNA sequence which is identical to TRD and MYC reference genes. A 184 bp DNA fragment is identical to MYC and TRD reference sequence. MYC gene chr 8:127741687 to 127741825 is continued with 45bp sequence on chr 14: 22423917 to 22423962 on TRD locus.

**Microscopic detection**

**Figure 9:**
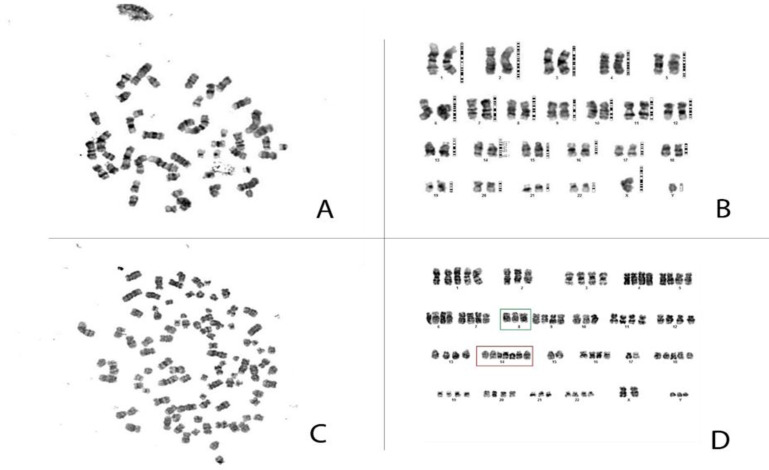
Chromosomal abnormality is a common feature in lymphoblastic cell lines. Microscopic analysis of SKW3 cell line showed cell polyploidy. There was no evidence of t (8;14)(q24;q11) MYC /TRD .This could be due to small range of chromosomal translocation in this cell line which occurs under the detection resolution limit. A-B represent a metaphase without polyploidy while C-D represent a metaphase with polyploidy.

There was no evidence regarding translocation or inversion in cell spreads. As expected, there were significant signs for chromosomal polyploidy in this cell line shown in Figure 9(C-D). The karyotyping procedure was repeated for several times in order to certify that there is no technical error. The cell source was cultured each time in order to reach the best dividing cells with suitable metaphases using karyotyping special medium (Chang Medium BMC, Cat. Number 91004). 

## Discussion

 Translocations with the involvement of MYC oncogene located at 8q24 can be different from patient to patient^   18 ^. In many cases, lymphoid malignancies occur as a result of errors in class switch recombination (CSR) or V(D)J recombination. The translocations caused by inadvisable V(D)J recombination juxtapose a proto-oncogene to a locus codes for T-cell receptor or an immunoglobulin. Therefore, the proto-oncogene becomes activated due to its proximity to T-cell receptor regulatory regions^   19 ^. The TCR translocations involve the TCRα/δ locus at chromosome 14q11 or TCRβ at chromosome 7q34 predominantly. The different mechanisms mediated by V(D)J recombination may lead to different fragments sequences, breakpoint location and clustering that make the PCR method unable to detect these breakpoints^   20 ^ . Approximately 7% of childhood and 12% of adult T-ALL are due to TCRA/D loci translocation ^   4 ^. The MYC transcript overexpression shows a great flexibility based on breakpoint cluster location. This event may result in limitation in terms of detection through molecular diagnostic methods. The chromosomal translocations have been appeared to be the most relevant event in hematological malignancies^   21 ^ . In this study, detection of MYC /TRD translocation was performed on SKW3 T-cells by using chromosome conformation capture assay. Results support the incidence of t(8;14) in these cells strongly which states this method is a promising technique in detection of chromosomal abnormalities. 

The results of inverse PCR on genomic DNA showed no specific bands in gel electrophoresis. This could be due to the long distance between two loci of interest compared to fixed 3C library. The cross-linked fragments from same or different chromosomes with suitable proximity increased the primer annealing chance in PCR amplification. The sizes of the fragments were different in each SKW3 3C library as a result of various folding and chromatin looping in library preparation. The different chromatin folding leads to different digestion and ligation products and variant fragment sizes in PCR amplification. In order to detect the juxtaposed sequence, we performed the translocation PCR using specific primers. As we mentioned, the “Tra4” PCR product was not a result of proximity and ligation of the fragments and it was contained no restriction site for EcoRI. This result indicated that the MYC /TRD is an intrinsic rearrangement in SKW3 cell line. The stained chromosomes of SKW3 cell line were investigated regarding appropriate timing. There was no evidence of translocation or inversion in any cell spreads. This result was supported by our knowledge on the nature of c-MYC/TRD translocation. As we discussed, the juxtaposed fragments are under the resolution detection range and the microscopic- based methods are unable to detect this small cluster of juxtaposed fragments. The juxtaposed fragment which we detected in this study is 184 bp which clearly is undetectable through karyotyping^   22 ^. Also, cytogenetic studies like chromosome karyotyping and metaphase FISH can be used only on dividing cells which can be difficult to obtain. On the other hand, only small range of chromosomal translocations can be detected through RT-PCR or conventional PCR due to the variant range of translocated segments next to other genes that could diverse the breakpoints. Although FISH is the most common laboratory and clinical method, it relies on technicians’ training in scoring the rearrangements through fluorescence microscopy. It requires specific expensive probes which are fully designed and purchased for that specific translocation. However, in cases with juxta- positioning or de-novo chromosomal rearrangements, it may lose its value and specificity. The Conventional PCR is a suitable method for detecting translocations such as bcr-abl t(9;22) and t(14;18)^   23 ^ .But PCR has its limitation regarding the chromosomal rearrangements with variant clustering incidence. This could be due to the requirement of multiple sets of specific primers for each translocated segment which is itself variable and their distance from the predicted region in each cell population based on the nature of hematopoietic cancers^   24 ^. Since, in many studies, 4C is limited to the description of long-range contacts with regions in cis or on other chromosomes. Frequently, a 4-cut restriction enzyme digestion was used to create smaller fragments more detectable in PCR amplification ^   25 ^.In a recent study, 3C-PCR was used to capture a variable region 1.3 Mb upstream of a chromosomal rearrangement breakpoint in a balanced translocation. It was shown that the nested PCR approach amplified the derivative chromosome fragments exclusively and identified the same haplotype by Sanger sequencing as it was desired^   7 ^. However, in this study, we used a single 6 cut EcoRI digestion in order to make sure the least occurrence of cutting events in TRD cluster translocated fragments. Although the 3C -based method failed to detect cell regarding cell differences in genome topology, it could capture many contacting fragments close or juxtaposed to the locus of interest without requiring many specific primers or high costs of microarray or NGS technologies^   26 ^. These data can provide a clear understanding in terms of interaction between a single locus and transcription factors or regulatory elements proximate or translocated to the locus of interest. In the 4C method, the captured fragments are amplified in non-equal efficiencies due to their differences in GC contents and their sizes. However , as it was discussed, the method is affordable and as it was called in the study by Denker et al. , it is a one-to-all method^   11 ^. In our investigation, all data in our investigation, all data including self-ligation and ligation between adjacent restriction fragments have been eliminated. The remaining intra-chromosomal interaction between chr8-c-MYC and chr14-TRD was aligned next to reference genome extracted from NCBI database. The results strongly support the hypothesis of chr8-C-Myc and ch14-TRD proximity and the PCR amplification on genomic DNA and 3C library clarifies the t (8; 14) presence in SKW3 cell line. As demonstrated in this study, we used a cheap, high-throughput 3C-based method for detecting chromosomal rearrangement.

## CONCLUSION

 This study is the first report on detecting t (8; 14) MYC /TRD using 3C- based method. We showed that small-sized chromosomal rearrangements can be detectable by using a cheap PCR -based technique. The microscopic method was unable to detect this translocation in SKW3 cell line. This method could be a useful way in detecting unknown chromosomal breakpoints and genes proximity. As we discussed, TRD fragments juxta positioning may have variety from patient to patient and their enhancing effect on oncogene expression could be different due to the variant nature of gene clustering. Therefore, further studies are needed to be conducted on different TRD juxtaposed fragments and different chromosomal breakpoints in other cancers.
